# *N*′-(3-Ethoxy-2-hydroxybenzylidene)­benzenesulfonohydrazide

**DOI:** 10.1107/S1600536808007988

**Published:** 2008-03-29

**Authors:** Xi-Shi Tai, Yi-Min Feng, Fan-Yuan Kong

**Affiliations:** a Department of Chemistry and Chemical Engineering, Weifang University, Weifang 261061, People’s Republic of China

## Abstract

There are two mol­ecules in the asymmetric unit of the title compound, C_15_H_16_N_2_O_4_S, both of which are stabilized by an intra­molecular O—H⋯N hydrogen bond. Inter­molecular N—H⋯O hydrogen bonds lead to [101] chains of mol­ecules in the crystal structure.

## Related literature

For related literature, see: Tai *et al.* (2003 ▶).
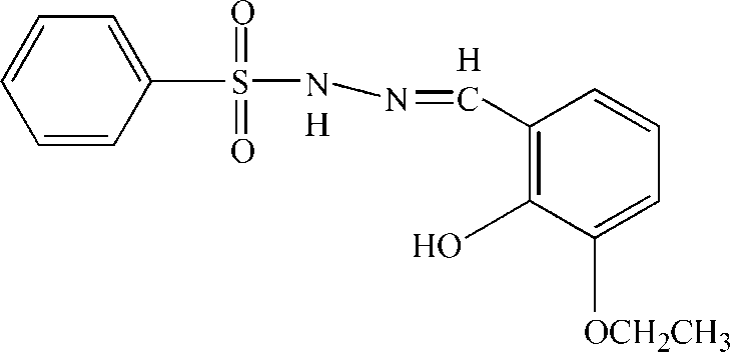


## Experimental

### 

#### Crystal data

C_15_H_16_N_2_O_4_S
*M* *_r_* = 320.36
Monoclinic, 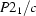

*a* = 10.3149 (10) Å
*b* = 39.935 (3) Å
*c* = 7.9832 (8) Å
β = 105.773 (2)°
*V* = 3164.7 (5) Å^3^
*Z* = 8
Mo *K*α radiation
μ = 0.22 mm^−1^
*T* = 298 (2) K
0.48 × 0.45 × 0.32 mm

#### Data collection

Bruker SMART CCD diffractometer
Absorption correction: multi-scan (*SADABS*; Bruker, 2000 ▶) *T*_min_ = 0.900, *T*_max_ = 0.932
16316 measured reflections
5576 independent reflections
3515 reflections with *I* > 2σ(*I*)
*R*_int_ = 0.047

#### Refinement

*R*[*F* ^2^ > 2σ(*F* ^2^)] = 0.074
*wR*(*F* ^2^) = 0.160
*S* = 1.09
5576 reflections
399 parameters
H-atom parameters constrained
Δρ_max_ = 0.29 e Å^−3^
Δρ_min_ = −0.41 e Å^−3^

### 

Data collection: *SMART* (Bruker, 2000 ▶); cell refinement: *SAINT* (Bruker, 2000 ▶); data reduction: *SAINT*; program(s) used to solve structure: *SHELXS97* (Sheldrick, 2008 ▶); program(s) used to refine structure: *SHELXL97* (Sheldrick, 2008 ▶); molecular graphics: *SHELXTL* (Sheldrick, 2008 ▶); software used to prepare material for publication: *SHELXTL*.

## Supplementary Material

Crystal structure: contains datablocks global, I. DOI: 10.1107/S1600536808007988/hb2710sup1.cif

Structure factors: contains datablocks I. DOI: 10.1107/S1600536808007988/hb2710Isup2.hkl

Additional supplementary materials:  crystallographic information; 3D view; checkCIF report

## Figures and Tables

**Table 1:** Hydrogen-bond geometry (Å, °)

*D*—H⋯*A*	*D*—H	H⋯*A*	*D*⋯*A*	*D*—H⋯*A*
O1—H1⋯N2	0.82	1.89	2.606 (5)	145
O5—H5⋯N4	0.82	1.87	2.591 (5)	146
N1—H1*A*⋯O5	0.90	2.14	2.948 (5)	149
N3—H3⋯O3^i^	0.90	2.11	2.912 (5)	147

Symmetry code: (i) 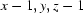.

**Fig. 1.**
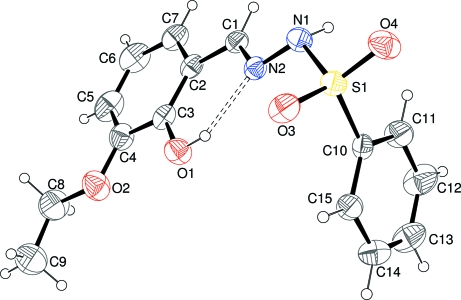
The C1-containing molecule in (I) showing 30% displacement ellipsoids for the non-hydrogen atoms. The intramolecular hydrogen bond is indicated by a double-dashed line.

## supplementary crystallographic information

### Comment

As part of our ongoing studies of aroylhydrazone ligands (Tai *et al.*, 2003), we now report the synthesis and structure of the title compound, (I), (Fig. 1).

There are two molecules in the asymmetric unit of (I), both of which are stabilised by an intramolecular O-H···N hydrogen bond (Table 1).  Then, intermolecular N-H···O hydrogen bonds lead to [101] chains of molecules in the crystal.

### Experimental

3-Ethoxysalicylaldehyde (3 mmol) was added to a solution of benzenesulfonyl hydrazide (3 mmol) in 10 ml of 95% ethanol.  The mixture was continuously stirred for 3 h at refluxing temperature, evaporating some ethanol, then, upon cooling, the solid product was collected by filtration and dried in vacuo (yield 78%). Colourless blocks of (I) were obtained by evaporation from a methanol solution after 3 days.

### Refinement

The H atoms were placed geometrically (C—H = 0.93–0.96 Å, N—H = 0.90 Å, O-H = 0.82Å) and refined as riding with *U*_iso_(H) = 1.2*U*_eq_(C, N) or 1.5*U*_eq_(methyl C, O).

### Crystal data

**Table:**

C_15_H_16_N_2_O_4_S	*F*_000_ = 1344
*M**_r_* = 320.36	*D*_x_ = 1.345 Mg m^−^^3^
Monoclinic, *P*2_1_/*c*	Mo *K*α radiation λ = 0.71073 Å
Hall symbol: -P 2ybc	Cell parameters from 3888 reflections
*a* = 10.3149 (10) Å	θ = 2.3–22.4º
*b* = 39.935 (3) Å	µ = 0.22 mm^−^^1^
*c* = 7.9832 (8) Å	*T* = 298 (2) K
β = 105.773 (2)º	Block, colourless
*V* = 3164.7 (5) Å^3^	0.48 × 0.45 × 0.32 mm
*Z* = 8	

### Data collection

**Table:**

Bruker SMART CCD diffractometer	5576 independent reflections
Radiation source: fine-focus sealed tube	3515 reflections with *I* > 2σ(*I*)
Monochromator: graphite	*R*_int_ = 0.047
*T* = 298(2) K	θ_max_ = 25.0º
ω scans	θ_min_ = 2.0º
Absorption correction: multi-scan(SADABS; Bruker, 2000)	*h* = −10→12
*T*_min_ = 0.900, *T*_max_ = 0.932	*k* = −47→47
16316 measured reflections	*l* = −8→9

### Refinement

**Table:**

Refinement on *F*^2^	Secondary atom site location: difference Fourier map
Least-squares matrix: full	Hydrogen site location: inferred from neighbouring sites
*R*[*F*^2^ > 2σ(*F*^2^)] = 0.074	H-atom parameters constrained
*wR*(*F*^2^) = 0.160	*w* = 1/[σ^2^(*F*_o_^2^) + (0.0121*P*)^2^ + 7.6148*P*] where *P* = (*F*_o_^2^ + 2*F*_c_^2^)/3
*S* = 1.09	(Δ/σ)_max_ < 0.001
5576 reflections	Δρ_max_ = 0.29 e Å^−^^3^
399 parameters	Δρ_min_ = −0.41 e Å^−^^3^
Primary atom site location: structure-invariant direct methods	Extinction correction: none

### Special details

**Table:**

Geometry. All e.s.d.'s (except the e.s.d. in the dihedral angle between two l.s. planes) are estimated using the full covariance matrix. The cell e.s.d.'s are taken into account individually in the estimation of e.s.d.'s in distances, angles and torsion angles; correlations between e.s.d.'s in cell parameters are only used when they are defined by crystal symmetry. An approximate (isotropic) treatment of cell e.s.d.'s is used for estimating e.s.d.'s involving l.s. planes.
Refinement. Refinement of *F*^2^ against ALL reflections. The weighted *R*-factor *wR* and goodness of fit *S* are based on *F*^2^, conventional *R*-factors *R* are based on *F*, with *F* set to zero for negative *F*^2^. The threshold expression of *F*^2^ > σ(*F*^2^) is used only for calculating *R*-factors(gt) *etc*. and is not relevant to the choice of reflections for refinement. *R*-factors based on *F*^2^ are statistically about twice as large as those based on *F*, and *R*- factors based on ALL data will be even larger.

### Fractional atomic coordinates and isotropic or equivalent isotropic displacement parameters (Å^2^)

**Table:**

	*x*	*y*	*z*	*U*_iso_*/*U*_eq_	
N1	0.8862 (4)	0.15474 (10)	0.5062 (5)	0.0505 (10)	
H1A	0.8190	0.1591	0.4102	0.061*	
N2	0.8858 (4)	0.12091 (10)	0.5385 (5)	0.0457 (10)	
N3	0.4135 (4)	0.16102 (10)	−0.1714 (5)	0.0555 (11)	
H3	0.3271	0.1542	−0.2033	0.067*	
N4	0.4452 (4)	0.16983 (10)	0.0019 (5)	0.0520 (10)	
O1	1.0083 (3)	0.06423 (9)	0.6356 (5)	0.0632 (10)	
H1	1.0025	0.0839	0.6056	0.095*	
O2	0.9949 (4)	0.00231 (10)	0.7310 (5)	0.0795 (12)	
O3	1.1327 (3)	0.16038 (9)	0.6246 (4)	0.0632 (10)	
O4	0.9990 (4)	0.20311 (8)	0.4304 (5)	0.0704 (11)	
O5	0.6248 (3)	0.18032 (8)	0.2945 (4)	0.0590 (9)	
H5	0.5955	0.1729	0.1956	0.088*	
O6	0.6879 (4)	0.20697 (10)	0.6006 (5)	0.0739 (11)	
O7	0.6420 (3)	0.13862 (10)	−0.1276 (5)	0.0732 (11)	
O8	0.4647 (3)	0.12903 (9)	−0.4027 (4)	0.0628 (10)	
S1	1.02564 (12)	0.16900 (3)	0.47484 (16)	0.0488 (3)	
S2	0.50608 (12)	0.13114 (4)	−0.21787 (17)	0.0560 (4)	
C1	0.7737 (5)	0.10720 (13)	0.5375 (6)	0.0490 (12)	
H1C	0.6947	0.1197	0.5077	0.059*	
C2	0.7698 (5)	0.07232 (13)	0.5827 (6)	0.0490 (12)	
C3	0.8843 (5)	0.05248 (13)	0.6323 (6)	0.0524 (13)	
C4	0.8766 (6)	0.01893 (14)	0.6801 (7)	0.0643 (15)	
C5	0.7519 (7)	0.00549 (16)	0.6748 (8)	0.084 (2)	
H5A	0.7448	−0.0169	0.7023	0.100*	
C6	0.6378 (7)	0.02532 (17)	0.6289 (9)	0.0834 (19)	
H6	0.5550	0.0162	0.6301	0.100*	
C7	0.6444 (5)	0.05806 (15)	0.5819 (7)	0.0646 (15)	
H7	0.5664	0.0709	0.5494	0.078*	
C8	0.9923 (7)	−0.03169 (15)	0.7840 (10)	0.094 (2)	
H8A	0.9388	−0.0451	0.6888	0.113*	
H8B	0.9529	−0.0333	0.8811	0.113*	
C9	1.1336 (8)	−0.04390 (16)	0.8364 (10)	0.106 (2)	
H9A	1.1737	−0.0407	0.7422	0.158*	
H9B	1.1347	−0.0673	0.8644	0.158*	
H9C	1.1839	−0.0316	0.9365	0.158*	
C10	1.0519 (4)	0.14629 (11)	0.2984 (6)	0.0446 (11)	
C11	0.9513 (5)	0.14558 (15)	0.1440 (7)	0.0672 (16)	
H11	0.8698	0.1564	0.1344	0.081*	
C12	0.9733 (7)	0.12869 (19)	0.0053 (8)	0.093 (2)	
H12	0.9058	0.1279	−0.0991	0.111*	
C13	1.0929 (7)	0.11296 (17)	0.0181 (8)	0.087 (2)	
H13	1.1065	0.1014	−0.0770	0.104*	
C14	1.1931 (6)	0.11413 (15)	0.1711 (8)	0.0729 (17)	
H14	1.2752	0.1037	0.1791	0.087*	
C15	1.1724 (5)	0.13063 (13)	0.3130 (7)	0.0567 (13)	
H15	1.2397	0.1311	0.4177	0.068*	
C16	0.3577 (5)	0.18495 (12)	0.0593 (7)	0.0536 (13)	
H16	0.2712	0.1881	−0.0130	0.064*	
C17	0.3915 (5)	0.19724 (11)	0.2357 (7)	0.0475 (12)	
C18	0.5232 (5)	0.19556 (11)	0.3449 (6)	0.0487 (12)	
C19	0.5557 (5)	0.20971 (12)	0.5107 (7)	0.0543 (13)	
C20	0.4553 (6)	0.22539 (13)	0.5660 (8)	0.0709 (16)	
H20	0.4764	0.2353	0.6754	0.085*	
C21	0.3247 (6)	0.22661 (14)	0.4621 (9)	0.0739 (17)	
H21	0.2576	0.2365	0.5031	0.089*	
C22	0.2943 (5)	0.21326 (13)	0.2996 (8)	0.0651 (16)	
H22	0.2064	0.2148	0.2290	0.078*	
C23	0.7310 (7)	0.22377 (17)	0.7650 (9)	0.094 (2)	
H23A	0.6864	0.2142	0.8464	0.113*	
H23B	0.7086	0.2474	0.7510	0.113*	
C24	0.8777 (8)	0.21949 (18)	0.8307 (9)	0.114 (3)	
H24A	0.8991	0.1960	0.8392	0.171*	
H24B	0.9086	0.2296	0.9437	0.171*	
H24C	0.9211	0.2300	0.7523	0.171*	
C25	0.4610 (5)	0.09376 (13)	−0.1326 (6)	0.0530 (13)	
C26	0.3513 (5)	0.07603 (15)	−0.2264 (8)	0.0721 (17)	
H26	0.3004	0.0840	−0.3335	0.087*	
C27	0.3164 (6)	0.04625 (16)	−0.1614 (10)	0.0851 (19)	
H27	0.2428	0.0340	−0.2254	0.102*	
C28	0.3898 (8)	0.03494 (18)	−0.0040 (11)	0.091 (2)	
H28	0.3668	0.0149	0.0397	0.109*	
C29	0.4971 (9)	0.05293 (19)	0.0901 (9)	0.098 (2)	
H29	0.5459	0.0452	0.1989	0.118*	
C30	0.5346 (6)	0.08246 (16)	0.0265 (8)	0.0758 (17)	
H30	0.6088	0.0945	0.0908	0.091*	

### Atomic displacement parameters (Å^2^)

**Table:**

	*U*^11^	*U*^22^	*U*^33^	*U*^12^	*U*^13^	*U*^23^
N1	0.034 (2)	0.060 (3)	0.053 (3)	0.0022 (18)	0.0051 (18)	0.002 (2)
N2	0.042 (2)	0.055 (3)	0.040 (2)	−0.0004 (18)	0.0097 (18)	0.0001 (18)
N3	0.039 (2)	0.063 (3)	0.057 (3)	0.003 (2)	0.001 (2)	0.000 (2)
N4	0.042 (2)	0.056 (3)	0.052 (3)	0.002 (2)	0.003 (2)	0.001 (2)
O1	0.046 (2)	0.064 (2)	0.079 (3)	−0.0032 (17)	0.0152 (18)	0.0077 (19)
O2	0.084 (3)	0.061 (2)	0.094 (3)	−0.002 (2)	0.023 (2)	0.012 (2)
O3	0.0392 (18)	0.097 (3)	0.048 (2)	−0.0035 (18)	0.0020 (16)	−0.0088 (19)
O4	0.069 (2)	0.045 (2)	0.100 (3)	−0.0005 (18)	0.029 (2)	−0.001 (2)
O5	0.051 (2)	0.071 (2)	0.051 (2)	0.0181 (17)	0.0067 (17)	−0.0066 (17)
O6	0.075 (3)	0.082 (3)	0.057 (2)	0.013 (2)	0.005 (2)	−0.020 (2)
O7	0.0317 (18)	0.101 (3)	0.079 (3)	−0.0013 (19)	0.0015 (18)	−0.004 (2)
O8	0.054 (2)	0.087 (3)	0.045 (2)	0.0002 (19)	0.0093 (16)	0.0056 (18)
S1	0.0385 (6)	0.0533 (8)	0.0516 (8)	−0.0008 (6)	0.0072 (6)	−0.0018 (6)
S2	0.0374 (7)	0.0746 (9)	0.0517 (8)	0.0028 (6)	0.0049 (6)	0.0025 (7)
C1	0.037 (3)	0.069 (3)	0.041 (3)	0.003 (2)	0.009 (2)	0.000 (2)
C2	0.045 (3)	0.063 (3)	0.039 (3)	−0.012 (2)	0.012 (2)	−0.007 (2)
C3	0.055 (3)	0.062 (3)	0.043 (3)	−0.012 (3)	0.018 (2)	−0.005 (2)
C4	0.073 (4)	0.059 (4)	0.063 (4)	−0.008 (3)	0.021 (3)	0.000 (3)
C5	0.100 (5)	0.069 (4)	0.092 (5)	−0.036 (4)	0.044 (4)	−0.010 (4)
C6	0.067 (4)	0.094 (5)	0.094 (5)	−0.036 (4)	0.030 (4)	−0.016 (4)
C7	0.055 (3)	0.078 (4)	0.064 (4)	−0.017 (3)	0.021 (3)	−0.009 (3)
C8	0.123 (6)	0.057 (4)	0.113 (6)	0.000 (4)	0.050 (5)	0.006 (4)
C9	0.135 (7)	0.071 (5)	0.118 (6)	0.023 (4)	0.045 (5)	0.008 (4)
C10	0.041 (3)	0.049 (3)	0.042 (3)	−0.004 (2)	0.009 (2)	0.009 (2)
C11	0.048 (3)	0.092 (4)	0.056 (4)	0.006 (3)	0.004 (3)	0.000 (3)
C12	0.083 (5)	0.140 (6)	0.050 (4)	0.000 (5)	0.010 (3)	−0.020 (4)
C13	0.092 (5)	0.111 (6)	0.065 (4)	−0.007 (4)	0.034 (4)	−0.023 (4)
C14	0.059 (4)	0.079 (4)	0.086 (5)	0.003 (3)	0.028 (3)	−0.008 (4)
C15	0.045 (3)	0.070 (3)	0.051 (3)	0.002 (3)	0.008 (2)	0.003 (3)
C16	0.039 (3)	0.045 (3)	0.074 (4)	0.003 (2)	0.011 (3)	0.011 (3)
C17	0.042 (3)	0.037 (3)	0.066 (3)	−0.001 (2)	0.017 (2)	0.004 (2)
C18	0.051 (3)	0.040 (3)	0.058 (3)	0.008 (2)	0.019 (3)	0.003 (2)
C19	0.054 (3)	0.045 (3)	0.066 (4)	0.001 (2)	0.018 (3)	0.000 (3)
C20	0.091 (5)	0.054 (3)	0.078 (4)	0.002 (3)	0.041 (4)	−0.011 (3)
C21	0.078 (4)	0.056 (4)	0.104 (5)	0.000 (3)	0.052 (4)	−0.012 (3)
C22	0.048 (3)	0.050 (3)	0.103 (5)	−0.001 (2)	0.031 (3)	0.004 (3)
C23	0.092 (5)	0.099 (5)	0.082 (5)	0.009 (4)	0.008 (4)	−0.036 (4)
C24	0.133 (7)	0.104 (6)	0.084 (5)	−0.003 (5)	−0.007 (5)	−0.032 (4)
C25	0.042 (3)	0.068 (3)	0.047 (3)	0.017 (2)	0.010 (2)	0.006 (3)
C26	0.047 (3)	0.084 (4)	0.081 (4)	0.001 (3)	0.010 (3)	0.023 (3)
C27	0.064 (4)	0.079 (5)	0.116 (6)	0.002 (3)	0.030 (4)	0.018 (4)
C28	0.104 (6)	0.077 (5)	0.107 (6)	0.024 (4)	0.057 (5)	0.024 (4)
C29	0.136 (7)	0.093 (6)	0.063 (4)	0.038 (5)	0.024 (5)	0.024 (4)
C30	0.080 (4)	0.077 (4)	0.061 (4)	0.016 (3)	0.002 (3)	0.000 (3)

### Geometric parameters (Å, °)

**Table:**

N1—N2	1.375 (5)	C10—C15	1.368 (6)
N1—S1	1.628 (4)	C10—C11	1.379 (6)
N1—H1A	0.8996	C11—C12	1.367 (8)
N2—C1	1.278 (5)	C11—H11	0.9300
N3—N4	1.378 (5)	C12—C13	1.363 (8)
N3—S2	1.633 (4)	C12—H12	0.9300
N3—H3	0.8998	C13—C14	1.370 (8)
N4—C16	1.271 (6)	C13—H13	0.9300
O1—C3	1.355 (5)	C14—C15	1.376 (7)
O1—H1	0.8187	C14—H14	0.9300
O2—C4	1.351 (6)	C15—H15	0.9300
O2—C8	1.425 (6)	C16—C17	1.442 (7)
O3—S1	1.432 (3)	C16—H16	0.9300
O4—S1	1.416 (3)	C17—C22	1.398 (7)
O5—C18	1.364 (5)	C17—C18	1.402 (6)
O5—H5	0.8202	C18—C19	1.394 (7)
O6—C19	1.362 (6)	C19—C20	1.381 (7)
O6—C23	1.433 (6)	C20—C21	1.378 (8)
O7—S2	1.423 (3)	C20—H20	0.9300
O8—S2	1.422 (3)	C21—C22	1.358 (8)
S1—C10	1.757 (5)	C21—H21	0.9300
S2—C25	1.755 (5)	C22—H22	0.9300
C1—C2	1.442 (7)	C23—C24	1.470 (9)
C1—H1C	0.9300	C23—H23A	0.9700
C2—C3	1.388 (7)	C23—H23B	0.9700
C2—C7	1.411 (6)	C24—H24A	0.9600
C3—C4	1.401 (7)	C24—H24B	0.9600
C4—C5	1.384 (8)	C24—H24C	0.9600
C5—C6	1.383 (9)	C25—C30	1.368 (7)
C5—H5A	0.9300	C25—C26	1.372 (7)
C6—C7	1.367 (8)	C26—C27	1.384 (8)
C6—H6	0.9300	C26—H26	0.9300
C7—H7	0.9300	C27—C28	1.356 (9)
C8—C9	1.485 (9)	C27—H27	0.9300
C8—H8A	0.9700	C28—C29	1.362 (9)
C8—H8B	0.9700	C28—H28	0.9300
C9—H9A	0.9600	C29—C30	1.380 (9)
C9—H9B	0.9600	C29—H29	0.9300
C9—H9C	0.9600	C30—H30	0.9300
			
N2—N1—S1	114.8 (3)	C13—C12—C11	120.9 (6)
N2—N1—H1A	108.0	C13—C12—H12	119.6
S1—N1—H1A	108.0	C11—C12—H12	119.6
C1—N2—N1	118.0 (4)	C12—C13—C14	119.9 (6)
N4—N3—S2	114.9 (3)	C12—C13—H13	120.0
N4—N3—H3	107.9	C14—C13—H13	120.0
S2—N3—H3	108.1	C13—C14—C15	120.2 (6)
C16—N4—N3	119.0 (4)	C13—C14—H14	119.9
C3—O1—H1	109.4	C15—C14—H14	119.9
C4—O2—C8	118.0 (5)	C10—C15—C14	119.3 (5)
C18—O5—H5	109.5	C10—C15—H15	120.4
C19—O6—C23	117.5 (4)	C14—C15—H15	120.4
O4—S1—O3	119.6 (2)	N4—C16—C17	120.4 (4)
O4—S1—N1	104.8 (2)	N4—C16—H16	119.8
O3—S1—N1	107.5 (2)	C17—C16—H16	119.8
O4—S1—C10	110.9 (2)	C22—C17—C18	117.7 (5)
O3—S1—C10	107.1 (2)	C22—C17—C16	120.3 (5)
N1—S1—C10	106.1 (2)	C18—C17—C16	121.8 (4)
O8—S2—O7	121.0 (2)	O5—C18—C19	117.1 (4)
O8—S2—N3	104.5 (2)	O5—C18—C17	122.0 (4)
O7—S2—N3	106.7 (2)	C19—C18—C17	120.9 (5)
O8—S2—C25	108.4 (2)	O6—C19—C20	126.7 (5)
O7—S2—C25	108.1 (2)	O6—C19—C18	114.5 (4)
N3—S2—C25	107.5 (2)	C20—C19—C18	118.7 (5)
N2—C1—C2	120.0 (4)	C21—C20—C19	121.3 (6)
N2—C1—H1C	120.0	C21—C20—H20	119.4
C2—C1—H1C	120.0	C19—C20—H20	119.4
C3—C2—C7	118.6 (5)	C22—C21—C20	119.7 (5)
C3—C2—C1	122.9 (4)	C22—C21—H21	120.2
C7—C2—C1	118.5 (5)	C20—C21—H21	120.2
O1—C3—C2	122.2 (5)	C21—C22—C17	121.7 (5)
O1—C3—C4	116.7 (5)	C21—C22—H22	119.1
C2—C3—C4	121.1 (5)	C17—C22—H22	119.1
O2—C4—C5	125.1 (6)	O6—C23—C24	107.8 (5)
O2—C4—C3	116.0 (5)	O6—C23—H23A	110.1
C5—C4—C3	118.9 (6)	C24—C23—H23A	110.1
C6—C5—C4	120.2 (6)	O6—C23—H23B	110.1
C6—C5—H5A	119.9	C24—C23—H23B	110.1
C4—C5—H5A	119.9	H23A—C23—H23B	108.5
C7—C6—C5	121.2 (6)	C23—C24—H24A	109.5
C7—C6—H6	119.4	C23—C24—H24B	109.5
C5—C6—H6	119.4	H24A—C24—H24B	109.5
C6—C7—C2	119.9 (6)	C23—C24—H24C	109.5
C6—C7—H7	120.0	H24A—C24—H24C	109.5
C2—C7—H7	120.0	H24B—C24—H24C	109.5
O2—C8—C9	107.5 (5)	C30—C25—C26	120.3 (5)
O2—C8—H8A	110.2	C30—C25—S2	120.1 (4)
C9—C8—H8A	110.2	C26—C25—S2	119.6 (4)
O2—C8—H8B	110.2	C25—C26—C27	119.9 (6)
C9—C8—H8B	110.2	C25—C26—H26	120.0
H8A—C8—H8B	108.5	C27—C26—H26	120.0
C8—C9—H9A	109.5	C28—C27—C26	119.8 (7)
C8—C9—H9B	109.5	C28—C27—H27	120.1
H9A—C9—H9B	109.5	C26—C27—H27	120.1
C8—C9—H9C	109.5	C27—C28—C29	120.1 (7)
H9A—C9—H9C	109.5	C27—C28—H28	120.0
H9B—C9—H9C	109.5	C29—C28—H28	120.0
C15—C10—C11	120.8 (5)	C28—C29—C30	121.0 (6)
C15—C10—S1	120.1 (4)	C28—C29—H29	119.5
C11—C10—S1	119.0 (4)	C30—C29—H29	119.5
C12—C11—C10	118.9 (5)	C25—C30—C29	118.9 (6)
C12—C11—H11	120.5	C25—C30—H30	120.6
C10—C11—H11	120.5	C29—C30—H30	120.6
			
S1—N1—N2—C1	−169.4 (3)	C12—C13—C14—C15	1.2 (10)
S2—N3—N4—C16	−159.3 (4)	C11—C10—C15—C14	0.3 (8)
N2—N1—S1—O4	175.3 (3)	S1—C10—C15—C14	−177.3 (4)
N2—N1—S1—O3	−56.5 (4)	C13—C14—C15—C10	−1.1 (9)
N2—N1—S1—C10	57.8 (4)	N3—N4—C16—C17	−174.3 (4)
N4—N3—S2—O8	−174.1 (3)	N4—C16—C17—C22	−178.7 (4)
N4—N3—S2—O7	−44.9 (4)	N4—C16—C17—C18	5.4 (7)
N4—N3—S2—C25	70.9 (4)	C22—C17—C18—O5	179.7 (4)
N1—N2—C1—C2	−175.6 (4)	C16—C17—C18—O5	−4.3 (7)
N2—C1—C2—C3	1.6 (7)	C22—C17—C18—C19	−0.6 (7)
N2—C1—C2—C7	179.4 (4)	C16—C17—C18—C19	175.4 (4)
C7—C2—C3—O1	179.6 (4)	C23—O6—C19—C20	−4.3 (8)
C1—C2—C3—O1	−2.5 (7)	C23—O6—C19—C18	174.1 (5)
C7—C2—C3—C4	0.3 (7)	O5—C18—C19—O6	1.4 (6)
C1—C2—C3—C4	178.1 (5)	C17—C18—C19—O6	−178.4 (4)
C8—O2—C4—C5	−0.1 (9)	O5—C18—C19—C20	179.9 (4)
C8—O2—C4—C3	178.8 (5)	C17—C18—C19—C20	0.1 (7)
O1—C3—C4—O2	2.5 (7)	O6—C19—C20—C21	179.7 (5)
C2—C3—C4—O2	−178.1 (5)	C18—C19—C20—C21	1.4 (8)
O1—C3—C4—C5	−178.5 (5)	C19—C20—C21—C22	−2.5 (9)
C2—C3—C4—C5	0.9 (8)	C20—C21—C22—C17	2.1 (8)
O2—C4—C5—C6	176.7 (6)	C18—C17—C22—C21	−0.6 (7)
C3—C4—C5—C6	−2.2 (9)	C16—C17—C22—C21	−176.6 (5)
C4—C5—C6—C7	2.4 (10)	C19—O6—C23—C24	−175.7 (5)
C5—C6—C7—C2	−1.1 (9)	O8—S2—C25—C30	151.3 (4)
C3—C2—C7—C6	−0.2 (8)	O7—S2—C25—C30	18.6 (5)
C1—C2—C7—C6	−178.1 (5)	N3—S2—C25—C30	−96.3 (4)
C4—O2—C8—C9	−178.9 (5)	O8—S2—C25—C26	−28.9 (5)
O4—S1—C10—C15	119.0 (4)	O7—S2—C25—C26	−161.7 (4)
O3—S1—C10—C15	−13.1 (5)	N3—S2—C25—C26	83.5 (5)
N1—S1—C10—C15	−127.7 (4)	C30—C25—C26—C27	−1.3 (8)
O4—S1—C10—C11	−58.7 (5)	S2—C25—C26—C27	179.0 (5)
O3—S1—C10—C11	169.2 (4)	C25—C26—C27—C28	0.9 (9)
N1—S1—C10—C11	54.6 (5)	C26—C27—C28—C29	0.3 (10)
C15—C10—C11—C12	0.5 (8)	C27—C28—C29—C30	−1.2 (11)
S1—C10—C11—C12	178.2 (5)	C26—C25—C30—C29	0.4 (8)
C10—C11—C12—C13	−0.5 (10)	S2—C25—C30—C29	−179.8 (5)
C11—C12—C13—C14	−0.4 (11)	C28—C29—C30—C25	0.8 (10)

### Hydrogen-bond geometry (Å, °)

**Table:**

*D*—H···*A*	*D*—H	H···*A*	*D*···*A*	*D*—H···*A*
O1—H1···N2	0.82	1.89	2.606 (5)	145
O5—H5···N4	0.82	1.87	2.591 (5)	146
N1—H1A···O5	0.90	2.14	2.948 (5)	149
N3—H3···O3^i^	0.90	2.11	2.912 (5)	147

Symmetry codes: (i) *x*−1, *y*, *z*−1.
